# Impact of Ileal Transposition Surgical Intervention on Antioxidant Status Measured in Liver Tissue of Obese Zucker Rats (Crl:ZUC-Lepr^fa^)

**DOI:** 10.1155/2018/7342451

**Published:** 2018-11-11

**Authors:** Bronisława Skrzep-Poloczek, Dominika Stygar, Tomasz Sawczyn, Ewa Romuk, Elżbieta Chełmecka, Jakub Poloczek, Agnieszka Dulska, Wojciech Kazura, Konrad W. Karcz

**Affiliations:** ^1^Department of Physiology, School of Medicine with the Division of Dentistry in Zabrze, Medical University of Silesia, Katowice, Poland; ^2^Department of Biochemistry, School of Medicine with the Division of Dentistry in Zabrze, Medical University of Silesia, Katowice, Poland; ^3^Department of Statistics, Department of Instrumental Analysis, School of Pharmacy with the Division of Laboratory Medicine in Sosnowiec, Medical University of Silesia, Katowice, Poland; ^4^Department of Rehabilitation, 3rd Specialist Hospital in Rybnik, Poland; ^5^Clinic of General, Visceral, Transplantation and Vascular Surgery, Hospital of the Ludwig Maximilian University, Munich, Germany

## Abstract

**Background:**

The main factor characteristic for low-grade systemic inflammation typical for obesity is oxidative stress (OS). Reactive oxygen species (ROS) production is higher and more increased in time in the obese patients than in lean subjects.

**Aims:**

To assess the effect of ileal transposition (IT) and sham types of bariatric procedures on the antioxidative systems in the liver tissue of Zucker rats (Crl:ZUC Lepr^fa^).

**Method:**

21 animals were divided into the experimental groups: control group (*n* = 7), sham group (*n* = 7), and IT group (*n* = 7). Sham and IT animals underwent selected surgery. The concentration of total antioxidant capacity (TAC), total antioxidant status (TOS), and activity of glutathione reductase (glutathione-disulfide reductase, GR, GSR), catalase (CAT), glutathione peroxidase (GPx), glutathione S-transferase (GST), and total superoxide dismutase activity (SOD) were assessed in liver tissue 3 months after surgery.

**Results:**

IT procedure significantly increased TAC when compared to sham and the control group. Animals after IT showed higher levels of TOS when compared to sham procedure. The total amount of TOS was similar in IT and control groups. GPx activity was increased in the groups submitted to the sham and IT surgery in relation to control. GR and CAT activities were significantly higher after IT in comparison to control and sham procedures. Total SOD and MnSOD were significantly higher in sham-operated animals in comparison to IT intervention and control groups.

**Conclusions:**

IT procedure had a positive impact on the diminishing of oxidative stress measured by TAC and TOS markers. The dynamic, adaptive, and protective mechanisms of enzymatic antioxidant systems were observed after the IT but not sham procedure. Nevertheless, 3 months after surgery, the midterm effect of bariatric surgery was observed, which might not fully balance the antioxidative response.

## 1. Introduction

One of the main factors characteristic for low-grade systemic inflammation and an immune activation characteristic for obesity is OS. ROS generation and inflammation increase more in time in obese patients than in lean subjects [1].

Bariatric surgery is, so far, the most efficient procedure for achieving significant and long-term body mass reduction in morbidly obese subjects [2]. It has been also showed to induce remission and significant improvement in type 2 diabetes mellitus and fatty liver disease. Bariatric surgery lowered mortality from cardiovascular risk and improved blood pressure, dyslipidemia, and dysglycemia [2–5]. Also, Roux-en-Y gastric bypass procedure (RYGB) was reported to significantly decrease the level of lipid peroxidation [4]. Ileal transposition (IT) is a type of jejunoileal bypass, notably reducing food intake [6]. Pathophysiologically, the remission of type 2 diabetes is associated with the active delivery of aliment to the terminal ileum [7]. Nevertheless, the comparative studies on OS after IT and sham procedures were not performed. In this work, we decided to use diabetic and obese Zucker rats (Crl:ZUC-Lepr^fa^, ZF) as an animal model of obesity, phenotypically close to human obesity [8].

The antioxidant defence under the conditions of OS caused by obesity considers the cumulative action of enzymatic and nonenzymatic antioxidant systems present in liver tissue [9]. The dynamic interaction between antioxidants as an answer to the IT and sham bariatric procedures is, therefore, the main aim of this study, hence giving a discernment into the sensitive balance between oxidative stress and antioxidant parameters 3 months after the surgery. This work is a new attempt at indicating the importance of comprehending the complex processes of the homeostatic control of antioxidants in liver tissue and its dynamic changes under oxidative stress development after IT and sham surgery.

## 2. Materials and Methods

### 2.1. Animals, Diet, and Study Design

The study was conducted according to the Guide for the Care and Use of Laboratory Animals (Directive 2010/63/EU) and the Ethics Committee of the Medical University of Silesia in Katowice, Poland. 21 male, obese12-week-old Zucker rats (Crl:ZUC Lepr^fa^, Charles River Breeding Laboratories, Wilmington, Mass, USA). The animals were kept under controlled conditions on a 12/12-hour light and dark cycle, in humidity 70 ± 1%, with unlimited access to water and rat food (Provimi Kliba AG, Kaiseraugst, Switzerland). Food contained 24% of protein, 4.9% of fat, 7% of crude ashes, 4.7% of crude fibre, lysine (13.6 g/kg), calcium (12 g/kg), methionine (4.5 g/kg), and phosphorus (8.3 g/kg).

**Figure 1:**
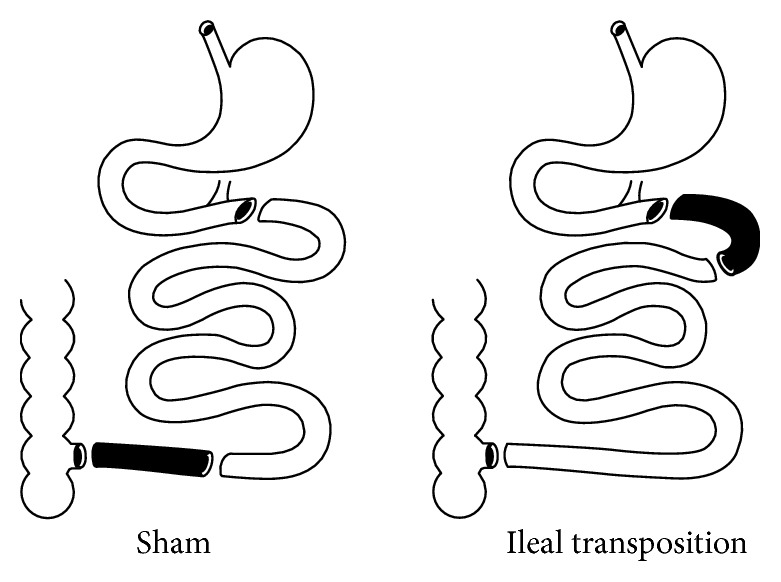
The scheme of the IT surgery.

After one week of acclimatisation, the animals were assigned to three experimental groups: control group CD (*n* = 7), sham group (*n* = 7), and IT group (*n* = 7). The sham and IT groups underwent two different types of surgery: sham, which is a control type of surgery, and IT (Figure 1). The control group was not included in any type of surgical intervention.

### 2.2. Ileal Transposition and Sham Surgery

The surgery procedures were previously described by Grüeneberger et al. [7]. Isoflurane 2% with oxygen flow at 2 l/min under spontaneous breathing was used to induce and maintain anaesthesia. After an abdominal midline incision, length 4–5 cm was performed and the Bauhin's valve was determined. 50% of the distal ileum was localized and transected. The ileal continuity was restored by an end-to-end extramucosal anastomosis using PDS 6/0 (Ethicon, Blue Ash, OH), excluding the transposed segment. Then, the ligament of Treitz was determined, and the jejunum was divided 5 cm aborally. The transposed segment of ileum was inserted in an isoperistaltic fashion, and two end-to-end anastomoses were performed. For control and sham surgery, transections were performed at all three analogous points. Anastomoses were completed correspondingly, nevertheless without IT (Figure 1). Fascia and skin closures were performed as a continuous suture using Monocryl 4/0 and Vicryl 4/0. After the surgery, all rats were kept on a liquid diet for 24 h (Nutrison, Nutricia, Poland).

### 2.3. Tissue Collection

Isoflurane 2% with oxygen flow at 2 l/min under spontaneous breathing was used to induce and maintain anaesthesia. At the end of the 12th week after surgery, corresponding to the 15th week of the experiment, the tissue of the liver was collected and the rats were euthanized. The muscle tissue (100 mg) was homogenized in 1 ml of a homogenising buffer. All samples were snap frozen in liquid nitrogen and stored at −80°C until further analysis. All experimental procedures were approved by the Ethical Committee for Animal Experimentation of the Medical University of Silesia (58/2014).

### 2.4. Oxidative Stress Marker Analysis

The total antioxidant capacity (TAC), total antioxidant status (TOS), and the activity of the following antioxidant enzymes: glutathione peroxidase (GPx), glutathione reductase (glutathione-disulfide reductase, GR, GSR), catalase (CAT), glutathione S-transferase (GST), total superoxide dismutase activity (SOD) in the liver tissue of the control, IT and Sham operated rats were determined.

### 2.5. Total Antioxidant Capacity (TAC)

TAC was measured using a commercial kit (Randox Co., England). The 2.2′azino-di-(3-ethylbenzothiazoline sulphonate) (ABTS) was incubated with a peroxidase (metmyoglobin) and hydrogen peroxide to produce the radical cation ABTS+, which has a relatively stable blue-green colour and was measured at 600 nm. The suppression of the colour was compared to the standard for TAC measurement assays (Trolox). The assay results are expressed as a Trolox equivalent (mmol/l). The inter- and intra-assay coefficients of variations (CV) were 1.1% and 3.8%, respectively.

### 2.6. Total Oxidative Status (TOS)

The method according to Erel [9] uses the oxidation of iron (II) ions to iron (III) ions in an acidic medium. Then iron (III) ions with xylene orange form a colourful complex ranging up to a blue-purple colouration. Absorption readings were taken with a 560 nm filter on the VICTOR-X3 from PerkinElmer. The TOS level was calculated from the calibration curve using H_2_O_2_ as the standard. Values are expressed in *μ*mol/l.

### 2.7. Oxidative Enzyme Analysis

#### 2.7.1. Glutathione Reductase Activity (EC 1.8.1.7)

GR enzymatic activity in the liver tissue was evaluated by a decrease in the concentration of NADPH in the samples using a GR buffer (200 mM sodium phosphate pH 7.5, 6.3 mm EDTA) and kinetic reading was performed at a wavelength of 340 nm for 10 minutes [10].

#### 2.7.2. Catalase Activity (EC 1.11.1.6)

The catalase activity in the liver tissue was measured using Aebi methods. Briefly, a 50 mM TRIS/HCl buffer, pH 7.4, and perhydrol were mixed with 50 *μ*l of tissue. After 10 seconds, the absorbance was read at *λ* = 240 nm, every 30 seconds for 2 minutes. The enzymatic activity was expressed in IU/mg protein [11].

#### 2.7.3. Glutathione Peroxidase Activity (EC 1.11.1.9)

To measure the activity of glutathione peroxidase, 40 mM sodium azide, GSH (diluted in 5% metaphosphoric acid), GR (GPx diluted in the buffer), NADPH (diluted with sodium bicarbonate 5%), and 0.5 mM tert-butyl were incubated in the liver tissue with a GPx buffer (100 mM potassium phosphate with 1 mM EDTA pH 7.7). The decay of NADPH concentration was evaluated for 10 minutes in a spectrophotometer, at 340 nm [12].

#### 2.7.4. Glutathione S-Transferase Activity (EC 2.5.1.18)

Transferase activity of glutathione S-transferase in the liver tissue was estimated by the kinetic method, previously described by Habig and Jakoby [13]. 1-Chloro-2,3-dinitrobenzene was used as a substrate and results are expressed in IU/g protein.

#### 2.7.5. Superoxide Dismutase Analysis (EC 1.15.1.1)

SOD isoenzymes' activity was determined with the use of the spectrophotometric method by Oyanagui [14]. KCN was used as the inhibitor of the CuZnSOD isoenzyme. CuZnSOD activity was calculated as the difference between total SOD activity and MnSOD activity. SOD activity was calculated against a blank probe (containing bidistilled water). Enzyme activity was expressed as nitrite units (NU) per mg of protein in tissue. One NU exhibits 50% inhibition of formation of nitrite ion under the method's condition [14].

#### 2.7.6. Protein Concentration

Protein concentration was determined by Lowry methods using bovine serum albumin as the standard [15].

## 3. Statistical Analysis

Statistical analysis was completed using STATISTICA 12.5 PL (StatSoft, Cracow, Poland.). A *p* value below 0.05 was accepted as statistically significant. All tests were two tailed. Interval data were expressed as a mean value ± standard deviation in the case of a normal distribution or as median/lower-upper quartile range in the case of data with skewed or nonnormal distribution. Distribution of variables was evaluated by the Shapiro-Wilk test, and the quantile-quantile plot. The homogeneity of variances was assessed by the Levene test. The two-way parametric ANOVA with post hoc contrast analysis, nonparametric Kruskal-Wallis test, or Mann-Whitney *U* test were used in order to compare the data. In the case of skewed data distribution, logarithmic transformation was done before analysis.

## 4. Results

**Figure 2:**
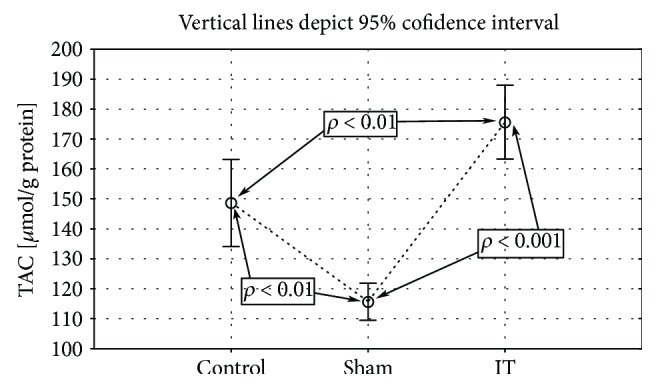
Mean values of TAC concentration in liver tissue of rats subjected to IT, sham operation type, and control group. Statistical significance was set at *p* < 0.001. The statistical significance IT, sham operation type vs. control group was shown, and individual points are connected for the reader's convenience.

**Figure 3:**
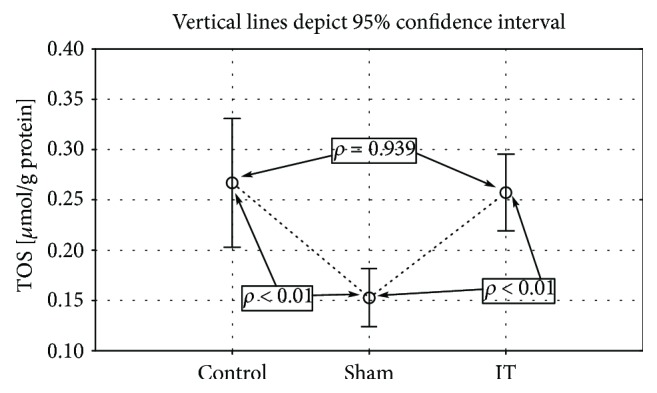
Mean values of TOS concentration in liver tissue of rats subjected to IT, sham operation type, and control group. Statistical significance was set at *p* < 0.05. The statistical significance IT, sham operation type vs. control group was shown, and individual points are connected for the reader's convenience.

The IT procedure increased significantly the TAC amount in comparison to the sham (*p* < 0.001) and control groups (*p* < 0.01). The lowest level of this parameter was observed in the sham-operated group (control vs. sham *p* < 0.01; Figure 2, Table 1). Also, the TOS parameter was significantly reduced in sham-operated rats. The control and IT groups showed a significantly higher TOS amount assessed in the liver tissue than sham-operated animals (control vs. sham *p* < 0.1, IT vs. sham *p* < 0.01; Figure 3, Table 1).

**Figure 4:**
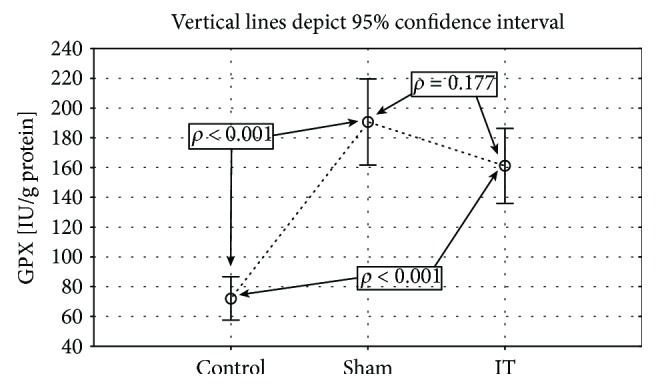
Mean values of GPx activity in liver tissue of rats subjected to IT, sham operation type, and control group. Statistical significance was set at *p* < 0.05. The statistical significance IT, sham operation type vs. control group was shown, and individual points are connected for the reader's convenience.

**Figure 5:**
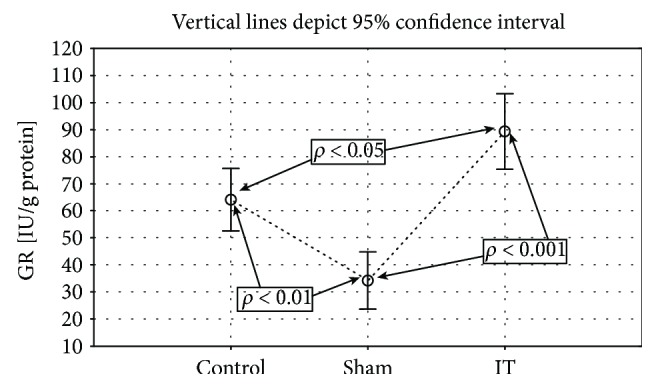
Mean values of GR activity in liver tissue of rats subjected to IT, sham operation type, and control group. Statistical significance was set at *p* < 0.05. The statistical significance IT, sham operation type vs. control group was shown, and individual points are connected for the reader's convenience.

**Figure 6:**
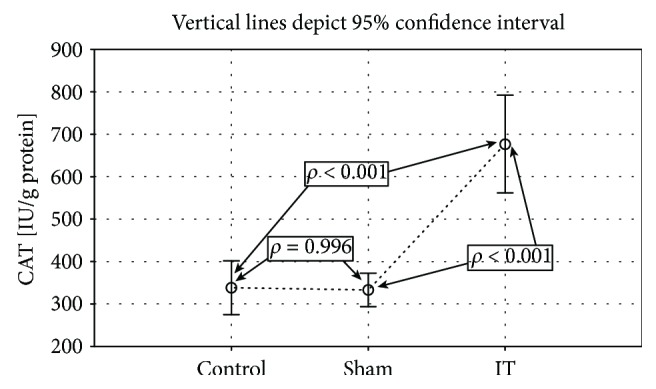
Mean values of CAT activity in liver tissue of rats subjected to IT, sham operation type, and control group. Statistical significance was set at *p* < 0.05. The statistical significance IT, sham operation type vs. control group was shown, and individual points are connected for the reader's convenience.

**Figure 7:**
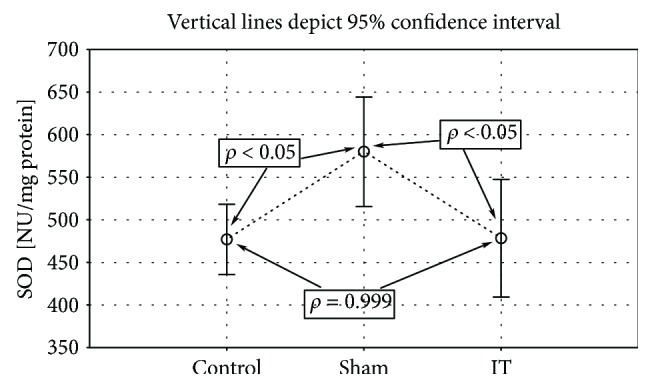
Mean values of Total SOD activity in liver tissue of rats subjected to IT, sham operation type, and control group. Statistical significance was set at *p* < 0.05. The statistical significance IT, sham operation type vs. control group was shown, and individual points are connected for the reader's convenience.

**Figure 8:**
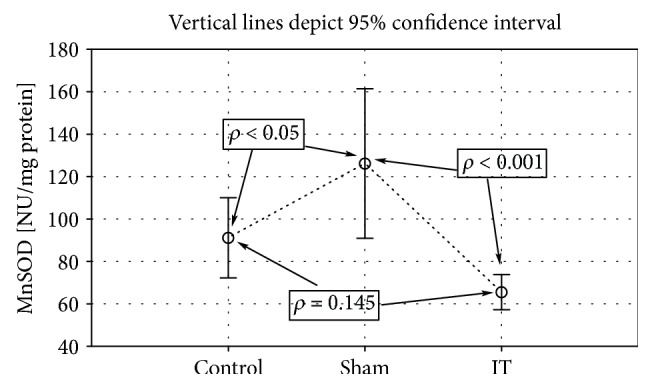
Mean values of MnSOD activity in liver tissue of rats subjected to IT, sham operation type, and control group. Statistical significance was set at *p* < 0.05. The statistical significance IT, sham operation type vs. control group was shown, and individual points are connected for the reader's convenience.

Significantly higher GPx activity was observed in the groups submitted to the IT and sham types of procedure when compared to the control group (control vs. sham *p* < 0.001, IT vs. sham *p* < 0.001; Figure 4, Table 1). The significant differences in the GR activity were found between the control, IT, and sham groups (control vs. sham *p* < 0.01, IT vs. control *p* < 0.05, IT vs. sham *p* < 0.001; Figure 5, Table 1). The GR activity was significantly higher in control and IT-operated animals when compared to animals submitted to the sham procedure. The IT procedure significantly influenced CAT activity in comparison to control and sham-operated animals (IT vs. control *p* < 0.001, IT vs. sham *p* < 0.001). After IT surgery, the CAT activity was twofold higher than in other studied animals (Figure 6, Table 1). Taking into consideration GST activity, there were no significant differences between the analysed groups observed (Table 1). The sham surgical intervention significantly increased total SOD activity when compared to control and IT-operated animals (control vs. sham *p* < 0.05, IT vs. sham *p* < 0.05; Figure 7, Table 1). A similar pattern for MnSOD activity was observed, where the highest activity of this enzyme was detected in sham-operated animals in comparison to the control and IT groups (control vs. sham *p* < 0.05, IT vs. sham *p* < 0.001; Figure 8, Table 1). CuZnSOD showed the same level of activity and no significant differences in the analysed groups: control, sham, and IT (Table 1).

## 5. Discussion

Bariatric surgery is currently one of the leading treatment options for morbid obesity, giving efficient and long-lasting results in reference to weight loss and glycemic control [16–18]. In this study, we have concentrated on the analysis of the influence of IT and sham procedures on the oxidative stress parameters measured in the liver tissue of animals 3 months after the IT and sham procedures: (i) the ileal transposition significantly increased TAC in comparison to sham-operated animals and the control group; (ii) animals after IT showed higher levels of TOS than sham-operated rats, and at the baseline, the total amount of TOS was congruous in IT and control study groups; (iii) GPx activity was increased in the groups submitted to the Sham and IT surgery in relation to control; (iv) GR and CAT were significantly higher after IT in comparison to the control and sham procedures; (vi) all analysed isoforms of SOD showed a similar pattern of activity in analysed groups. Total SOD and MnSOD were significantly higher in sham-operated animals in comparison to the IT intervention and control groups.

Obesity, a chronic disease, is interconnected with the augmentation of a broad range of health problems, which may lead to morbidity and mortality [19]. Under pathophysiological conditions such as overweight, T2DM, cardiovascular disease, and atherogenic processes, OS is induced and, successively, is associated with a defective production of adipokines, which further the progress of the metabolic syndrome [19, 20]. TAC is understood as the additive action of all the antioxidants present in the selected tissue; hence, it is an integrated factor rather than the ordinary sum of measurable antioxidants, strongly modified by OS [21–23]. According to Serafini and Del Rio [23], TAC should be understood as a “conception” rather than an analytical technique, an idea of total antioxidant efficiency reflecting the complex aspects of redox interactions. Plasma TAC values were observed to be decreased in patients with type 1 diabetes and type 2 diabetes in comparison to matched healthy controls [24–26]. Other human studies also reported higher levels of oxidative stress, despite excessive antioxidant capacity, in the plasma of patients with uncomplicated type 2 diabetes compared with healthy control subjects [27]. The TAC concentration was decreased under the condition of obesity, which was associated with systemic antioxidant defence and increased oxidative stress [28]. The significant differences in TAC between the IT, sham, and control liver samples in the present study suggest that reduced total ROS content in the IT group is more likely due to increased scavenging of free radicals and other toxic species. Our observed increase in TAC in IT animals compared to Sham and controls further fosters evidence proposing that reduced oxidative stress after an IT procedure may be the main contributor for ameliorating insulin resistance and the negative consequences of obesity conditions. The levels of different oxidant species may be analysed separately, but the measurements are demanding, costly, and require advanced techniques [29]. Since the analysis of various oxidant molecules independently is not practical and their oxidant effects are additive, the body's oxidant/antioxidant status can be ascertained by measuring the TOS [29]. Therefore, TAC and TOS are more precise markers of the oxidative and antioxidative status of individuals and TOS levels can be understood as a general indicator of oxidant molecules [29]. 3 months after a sham procedure, liver antioxidant systems seem to be able to neutralize only part of the ROS produced, which was observed in significantly reduced TOS in the sham group when compared to the IT and control groups. We suggest that stress connected with sham intervention, which is a kind of control procedure without therapeutic outcomes, was too high and the system did not restore TOS to levels observed in other control and IT groups. We believe that the IT procedure showed a beneficial effect on the TOS amount in comparison to sham but did not improve the TOS amount significantly as was visible for the TAC amount.

H_2_O_2_ is metabolised by CAT and GPx and in that way reduced to water and molecular oxygen. GPx reduces H_2_O_2_ and organic peroxides (ROO) while oxidising glutathione (GSH) [30]. Oxidised glutathione (GSSG) is subsequently reduced back to GSH by glutathione reductase (GR) in the presence of NADPH (or the corresponding alcohol (ROH)) and GSSG. The reduced form of GSH is a key intracellular, most abundant antioxidant, which conjugates with electrophiles and takes a part in the metabolism and detoxification of endogenous compounds as well as xenobiotics [30]. Changes in glutathione status have been broadly reported in oxidative stress-related disorders, but the present study is the first comprehensively investigating the enzymes of glutathione status such as GR and GPx in the liver tissue of diabetic and obese Zucker rats after IT and sham surgery. The special attention is focused on the changes in GPx activity, as the expression of the enzyme is proved to be increased under conditions of excessive ROS production [31]. The increased values of GPx in the liver tissue of the animals after the sham, but not IT procedure, may suggest that the disturbed liver glutathione status of rats might result from the enhancement of GSH consumption in the process catalysed by glutathione peroxidase. It appears feasible that the attenuation of oxidative stress accomplished by the IT treatment affects the expression of the GPx of glutathione metabolism, which has been proposed to be ROS sensitive [31]. Other human studies proved the correlation between visceral fat accumulation and the enhanced oxidative status, where there is a significant decrease of GPx activity in the subgroups of the highest BMI category and the highest quartiles of waist circumference (WC) [32, 33]. The positive, reductive impact of the IT procedure on the body weight of operated rats was already presented by Grüeneberger et al., and thus, we can assume that the antioxidant defence measured by GPx activity was significantly stronger in the IT but not in the sham group [7]. The control group, without any intervention, expressed the lowest level of GPx activity. The differences in the mobilization of the GPx/GR system in sham-operated animals may be understood as inefficiency or overloading of GR activity with the free radical formation when compared to the control and IT procedures. There was a significant increase in CAT enzymatic activity in IT-operated animals. This can be a positive effect of the bariatric procedure on the CAT antioxidative defence as it is known that CAT activity was found to be significantly diminished upon the increase of adipose tissue [19].

Under the conditions of long persisting obesity, the pool of antioxidant sources can be diminished, further affecting the activity of enzymes such as superoxide dismutase (SOD). In human subjects, the activity of SOD in obese individuals was significantly reduced in comparison to that in healthy subjects, intensifying the development of obesity-related health problems [19]. Obesity has been connected with an enhanced expression of NADPH oxidase and a restriction in the expression of various antioxidant proteins. In this study, which is an animal model of obesity, the total SOD and MnSOD measured in the sham group were significantly increased in comparison to the IT and control groups. The elevation of SOD activity may be understood as amplification of antioxidant capacity in sham animals and consequently a reduction in oxidative lesions. It is essential to indicate that an increase in total SOD, MnSOD, and CAT activities after bariatric surgery can be related to the time after surgery and reduction of adipose tissue. It is known that metabolic surgery improves the inflammatory response during the medium term after surgery. Human studies have found that oxidative stress was significantly reduced 6 months after bariatric surgery [19, 34, 35]. Murri et al. reported that 9 months after biliopancreatic diversion, the TAC and the activity of CAT and SOD did not change significantly during the study [36]. It is suggested that a long-term effect of bariatric surgery observed by reduced mass of adipocytes, and thus decline in the synthesis of molecules directly related to hypertrophic adipose tissue, might balance the antioxidant response.

## 6. Conclusions

We conclude that the IT procedure had a positive impact on the reduction of oxidative stress, measured by TAC and TOS parameters in the liver tissue of obese rats. The dynamic, adaptive, and protective mechanisms of enzymatic antioxidant systems were observed after the IT but not sham procedure. Nevertheless, 3 months after surgery, the midterm effect of bariatric surgery was observed, which might not fully balance the antioxidative response.

## Figures and Tables

**Table 1:** Antioxidant concentration and activity in liver tissue 3 months after IT surgery. Statistical significance was set at *p* < 0.05.

	Control	Sham	IT	*p*	*p *_1_ Control vs sham	*p *_2_ IT vs control	*p *_3_ IT vs sham
TAC (*μ*mol/g)	148.71 ± 15.71	115.63 ± 6.68	175.62 ± 17.29	**<0.001**	**<0.01**	**<0.01**	**<0.001**
TOS (*μ*mol/g)	0.27 ± 0.07	0.15 ± 0.03	0.26 ± 0.05	**<0.001**	**<0.01**	0.939	**<0.01**
GPx (IU/g)	72.02 ± 15.77	190.70 ± 31.32	161.21 ± 35.24	**<0.001**	**<0.001**	**<0.001**	0.177
GR (IU/g)	64.09 ± 27.74	34.22 ± 11.40	89.36 ± 19.50	**<0.001**	**<0.01**	**<0.05**	**<0.001**
CAT (IU/g)	338.38 ± 68.20	333.22 ± 42.85	676.43 ± 160.44	**<0.001**	0.996	**<0.001**	**<0.001**
Total SOD (NU/mg)	477.12 ± 44.51	579.82 ± 69.38	478.24 ± 96.53	**<0.05**	**<0.05**	0.999	**<0.05**
MnSOD (NU/mg)	91.09 ± 20.43	126.10 ± 38.10	65.49 ± 11.60	**<0.001**	**<0.05**	0.145	**<0.001**
GST (IU/g)	35.29 ± 1.63	40.31 ± 4.84	38.16 ± 6.46	0.179	—	—	—
CuZnSOD (NU/mg)	386.27 ± 47.40	439.41 ± 60.06	368.75 ± 71.10	0.086	—	—	—

Abbreviations: GPX: glutathione peroxidase; GR: glutathione reductase; CAT: catalase, SOD: total superoxide dismutase; MnSOD: Mn superoxide dismutase; ZnSOD: Zn superoxide dismutase; GST: glutathione S-transferase: TAC: total antioxidant capacity; TOS: total oxidative status.

## Data Availability

The data used to support the findings of this study are available from the corresponding author upon request.
